# Health‐care resource use and costs associated with diabetic and idiopathic gastroparesis: A claims analysis of the first 3 years following the diagnosis of gastroparesis

**DOI:** 10.1111/nmo.14366

**Published:** 2022-03-30

**Authors:** Yaozhu J. Chen, Wenxi Tang, Raluca Ionescu‐Ittu, Rajeev Ayyagari, Eric Wu, Susanna Y. Huh, Henry P. Parkman

**Affiliations:** ^1^ Takeda Development Center Americas, Inc. Cambridge Massachusetts USA; ^2^ Analysis Group, Inc. Boston Massachusetts USA; ^3^ 25139 Temple University Hospital Philadelphia Pennsylvania USA

**Keywords:** diabetic gastroparesis, gastroparesis, health‐care costs, health‐care resource utilization, idiopathic gastroparesis

## Abstract

**Background:**

Due to limited treatment options, many patients with diabetic gastroparesis (DG) or idiopathic gastroparesis (IG) experience inadequate symptom control resulting in increased health‐care resource utilization (HRU) and associated costs. We compared all‐cause HRU and health‐care costs over the 3 years after patients’ first gastroparesis diagnosis with that of matched controls without gastroparesis.

**Methods:**

Newly diagnosed adults with DG or IG were identified in Optum's de‐identified Clinformatics^®^ Data Mart Database (Q1‐2007 to Q1‐2019). Patients with DG/IG were matched 1:1 to controls using a mixed approach of exact matching and propensity score matching. The index date was the first gastroparesis diagnosis for cases or randomly selected for controls. All‐cause HRU and direct health‐care costs per person‐year (PPY) were compared between DG/IG cases and controls in Years 1–3 post‐index.

**Key Results:**

Demographics and comorbidities were balanced between patients with gastroparesis (*n* = 18,015 [DG]; *n* = 14,305 [IG]) and controls. In each of the Years 1–3 post‐index, patients with DG or IG had significantly higher annual HRU and costs versus controls (mean total cost differences PPY: DG Year 1 $34,885, Year 2 $28,071, Year 3 $25,606; IG Year 1 $23,176, Year 2 $16,627, Year 3 $14,396) (all *p* < 0.05). Across all 3 years, DG/IG cohorts had approximately twice the costs of controls. HRU and costs were highest in Year 1 post‐index for both DG and IG.

**Conclusions & Inferences:**

The economic burden of gastroparesis remains high several years after diagnosis, emphasizing the need for chronic treatment to effectively manage symptoms and consequently reduce the burden of this disorder.

## Graphical abstract

Healthcare resource use and costs associated with diabetic and idiopathic gastroparesis: A claims analysis of the first three years following the diagnosis of gastroparesis.

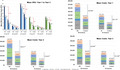

**Key Points**

- The aim of this retrospective US claims study was to quantify the direct medical burden of gastroparesis (all‐cause healthcare resource utilization and associated costs attributable to having gastroparesis).
- This study demonstrates the substantial economic burden associated with diabatic and idiopathic gastroparesis over each of the first three years after the initial diagnosis of gastroparesis.
- The results emphasize the need for chronic treatment to effectively manage symptoms and consequently reduce the burden of this disorder.

## 1 INTRODUCTION

Gastroparesis is a chronic disorder characterized by reduced stomach muscle motility and delayed gastric emptying without mechanical obstruction.^1^ The most commonly reported subtypes are idiopathic gastroparesis (IG) and diabetic gastroparesis (DG).^2^, ^3^ IG represents a prevalent etiology subgroup in which a cause cannot be identified.^4^, ^5^ DG is often associated with poorly controlled type I or type II diabetes and may occur with other diabetic complications and result in frequent hospitalizations.^6^, ^7^, ^8^ Regardless of type, symptoms of gastroparesis include nausea, vomiting, early satiety, bloating, upper abdominal pain, and post‐prandial fullness, which may negatively impact nutrition.^3^ In severe cases, patients may experience weight loss, malnutrition, dehydration, electrolyte imbalance, hypo‐ or hyperglycemic fluctuations among diabetics, and bezoar formation.^3^, ^9^ The overall prevalence of gastroparesis in the United States (US) was estimated at 16 per 100,000 persons during 1999–2014.^10^

While mild gastroparesis may be managed with non‐pharmacologic measures (e.g., dietary modification and hydration), patients with prolonged or moderate‐to‐severe gastroparesis symptoms are often managed with medications (e.g., prokinetics to improve gastric motility and antiemetics to reduce vomiting and nausea).^11^, ^12^, ^13^ The prokinetic metoclopramide is the only currently approved agent by the US Food and Drug Administration for the treatment of gastroparesis (DG only), but it carries a black box warning for tardive dyskinesia and other extrapyramidal symptoms.^13^ No medication is approved for gastroparesis in the European Union. Furthermore, other prokinetics or antiemetic treatment options have limited efficacy, and some are associated with side effects such as cardiovascular and extrapyramidal events.^14^, ^15^

Gastroparesis is also associated with a high economic burden and a negative impact on health‐related quality of life (HRQoL),^16^, ^17^, ^18^, ^19^ likely related to the lack of effective treatments for persistent, uncontrolled symptoms, and delayed diagnosis. Indeed, prior studies indicate that delays in diagnosis or misdiagnosis of gastroparesis are common in real‐world practice due to the overlap between its symptoms and those of other gastrointestinal disorders (e.g., functional dyspepsia and ulcer).^20^, ^21^, ^22^, ^23^, ^24^ Furthermore, patients with gastroparesis often have lower HRQoL with limited ability to perform daily activities due to uncontrolled symptoms.^17^, ^25^ Wadhwa et al. found that the number of inpatient admissions due to gastroparesis had risen significantly over time, contributing estimated costs of over $550 million in 2017 to the US health system.^16^ However, real‐world evidence on the health‐care resource utilization (HRU) and costs associated with gastroparesis remains limited, especially long‐term HRU and costs after the diagnosis of gastroparesis. Given the lack of effective treatments for gastroparesis,^14^, ^15^ the associated economic burden may be expected to manifest long term. Furthermore, prior studies have primarily focused on inpatient hospital admissions or emergency room (ER) visits and have not used matched patients without gastroparesis as controls to assess the additional costs and HRU associated with gastroparesis.^16^, ^17^, ^26^

To address this knowledge gap of the real‐world HRU and costs with gastroparesis, this study used a large US administrative claims database to quantify the direct medical burden of gastroparesis (all‐cause HRU and associated costs) among patients with DG or IG in the first 3 years following the initial gastroparesis diagnosis compared to matched controls without gastroparesis.

## 2 METHODS

### 2.1 Data source

This study used de‐identified data from the Optum Clinformatics Data Mart Database (Q1‐2007 to Q1‐2019), a commercial claims database including health‐care information on 15 million beneficiaries per year insured by commercial or Medicare Advantage plans in the US.^27^ This database, updated on regular basis, spans all 50 states and captures patients nationwide. Data elements include enrollment history, prescription, medical, and laboratory claims of beneficiaries. This study used anonymized claims data; thus, no institutional board review was required.

### 2.2 Selection of DG and IG cohorts and matched controls

**FIGURE 1:**
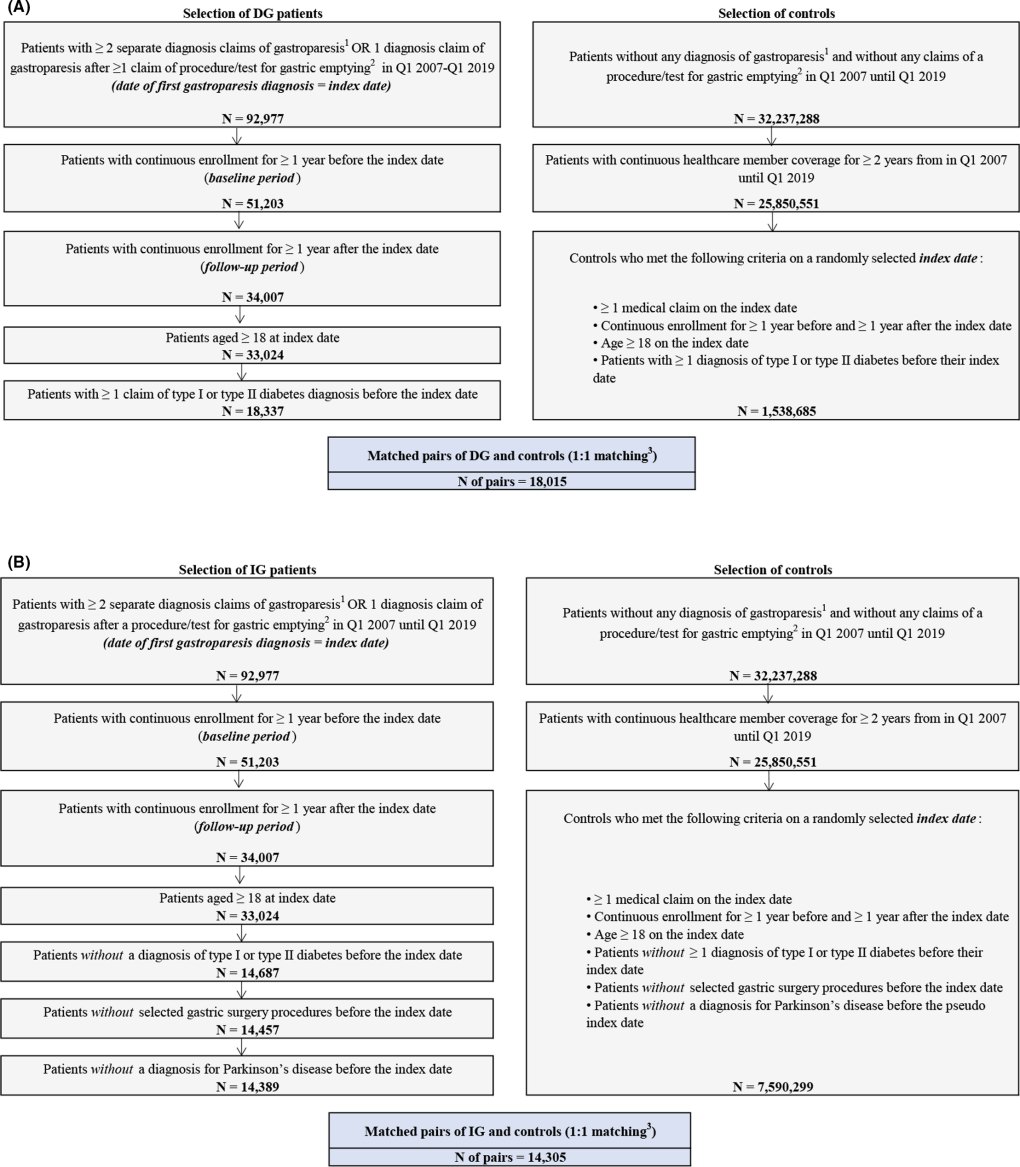
Sample Selection of (A) the DG Cohort and Matched Controls and (B) the IG Cohort and Matched Controls. Abbreviations: DG, diabetic gastroparesis; IG, idiopathic gastroparesis; Q, quarter. ^1^Diagnosis claims for gastroparesis were required to be incurred in an inpatient facility, an outpatient facility, or from professional services (excluding laboratory, radiology, and pathology) settings. ^2^For patients with only one gastroparesis claim, the gastric emptying procedure was required to be before the date of the gastroparesis claim. ^3^DG and IG potential controls were matched 1:1 to patients in the respective cohort using a mixed approach of exact matching on some covariates (age at index date, sex, and length of continuous enrollment) and propensity score matching

Adults (aged ≥18 years) newly diagnosed with DG or IG between January 1, 2007 and March 31, 2019 were identified in the Optum database (Figure 1). The date of the patient's first observed gastroparesis diagnosis during the study observation period was the *index date*. To capture patients newly diagnosed with gastroparesis, a 1‐year period without gastroparesis diagnosis was required prior to the index date (*baseline period*). By design, all patients were observed in the data for at least 1 year post‐index date and were included in the analyses of HRU and costs in the 1^st^ year after the gastroparesis diagnosis. The subsets of patients with ≥2 years and ≥3 years of continuous enrollment after the index date were included in the analyses for 2‐year and 3‐year follow‐up, respectively.

Patients newly diagnosed with gastroparesis who met the selection criteria (as shown in Figure 1A,B) were categorized into two mutually exclusive cohorts. The *DG cohort* included those with at least one diagnosis claim for type I or type II diabetes before the index date. The *IG cohort* included the remaining patients after excluding those with fundoplication or with a diagnosis of Parkinson's disease before the index date.

#### 2.2.1 Matching of controls with the DG and IG cohorts

Potential controls for the DG and IG cohorts were selected from the general population using similar criteria as those used for the DG and IG cohorts (Figure 1). For the potential controls, the index date was selected randomly from all dates with ≥1 medical claim. Controls for the DG cohort were required to have ≥1 diagnosis of type I or type II diabetes before their index date. DG controls and IG controls were matched 1:1 to the patients in the DG or IG cohorts, respectively, using a mixed approach of exact matching on key covariates (age at index date, sex, and years of continuous enrollment; same value required) and matching on propensity score using a 0.01 caliper (i.e., the maximum difference between matched subjects’ propensity scores).

For both cohorts, the propensity scores used for matching were derived from logistic models that included demographics (age, sex, race, payer type, and region of residence), socioeconomic characteristics (income and education), year of the index date, and all individual comorbidities included in the Charlson Comorbidity Index (CCI). For the DG cohort matching, the propensity score logistic models also included diabetes characteristics and diabetes complications (i.e., diabetes type, insulin use, HbA1c level, hypertension, stroke, angina, retinopathy, nephropathy, neuropathy, diabetic foot, and obesity^28^, ^29^, ^30^, ^31^, ^32^).

### 2.3 Measurements

Patient characteristics, measured during the baseline period, included all matching variables in the propensity score models and additional variables that may reflect the burden of gastroparesis pre‐diagnosis (Table 1). Comorbidities more commonly observed among patients with gastroparesis were identified according to the list reported by Nassar and Richter.^32^

**TABLE 1:** Baseline characteristics for the DG and IG cohorts and matched controls

	DG cohort and matched controls			IG cohort and matched controls		
	DG (*N* = 18,015)	Controls (*N* = 18,015)	Standard difference^a^	IG (*N* = 14,305)	Controls (*N* = 14,305)	Standard difference^a^
**MATCHING VARIABLES**						
**Demographics at index date**						
Age, mean (SD)	61.8 (13.4)	61.8 (13.4)	─	54.4 (17.5)	54.4 (17.5)	─
Females, *n* (%)	11,912 (66.1%)	11,912 (66.1%)	─	10,810 (75.6%)	10,810 (75.6%)	─
Region of residence, *n* (%)						
South	9532 (52.9%)	9498 (52.7%)	0.4%	7209 (50.4%)	7273 (50.8%)	−0.9%
Midwest	3269 (18.1%)	3223 (17.9%)	0.7%	2924 (20.4%)	2804 (19.6%)	2.1%
Northeast	1610 (8.9%)	1511 (8.4%)	2.0%	1277 (8.9%)	1219 (8.5%)	1.4%
West	3509 (19.5%)	3635 (20.2%)	−1.8%	2841 (19.9%)	2896 (20.2%)	−1.0%
Unknown	95 (0.5%)	148 (0.8%)	−3.6%	54 (0.4%)	113 (0.8%)	−5.4%
Race, *n* (%)						
Asian	333 (1.8%)	336 (1.9%)	−0.1%	225 (1.6%)	226 (1.6%)	−0.1%
Black	2739 (15.2%)	2645 (14.7%)	1.5%	1207 (8.4%)	1136 (7.9%)	1.8%
Hispanic	2143 (11.9%)	2103 (11.7%)	0.7%	1063 (7.4%)	1066 (7.5%)	−0.1%
White	9823 (54.5%)	9957 (55.3%)	−1.5%	9287 (64.9%)	9307 (65.1%)	−0.3%
Unknown	2977 (16.5%)	2974 (16.5%)	0.0%	2523 (17.6%)	2570 (18.0%)	−0.9%
**Index year, *n* (%)**						
2008–2011	5752 (31.9%)	6304 (35.0%)	−6.5%	5632 (39.4%)	5911 (41.3%)	−4.0%
2012–2015	6786 (37.7%)	7593 (42.1%)	−9.2%	5225 (36.5%)	5544 (38.8%)	−4.6%
2016–2018	5477 (30.4%)	4118 (22.9%)	17.1%	3448 (24.1%)	2850 (19.9%)	10.1%
**Full years of continuous enrollment post‐index** ^b^ **, *n* (%)**						
≥2 years	11,870 (65.9%)	11,870 (65.9%)	─	9857 (68.9%)	9857 (68.9%)	─
≥3 years	7838 (43.5%)	10,122 (56.2%)	─	6837 (47.8%)	8436 (59.0%)	─
**Socioeconomic characteristics**						
Income, *n* (%)						
<$40K	5538 (30.7%)	5605 (31.1%)	−0.8%	2923 (20.4%)	2878 (20.1%)	0.8%
$40K–$49K	1313 (7.3%)	1350 (7.5%)	−0.8%	829 (5.8%)	848 (5.9%)	−0.6%
$50K–$59K	1266 (7.0%)	1223 (6.8%)	0.9%	857 (6.0%)	890 (6.2%)	−1.0%
$60K–$74K	1607 (8.9%)	1641 (9.1%)	−0.7%	1276 (8.9%)	1270 (8.9%)	0.1%
$75K–$99K	1995 (11.1%)	2042 (11.3%)	−0.8%	1827 (12.8%)	1891 (13.2%)	−1.3%
$100K+	2433 (13.5%)	2490 (13.8%)	−0.9%	3694 (25.8%)	3704 (25.9%)	−0.2%
Unknown	3863 (21.4%)	3664 (20.3%)	2.7%	2899 (20.3%)	2824 (19.7%)	1.3%
Education, *n* (%)						
Less than 12th grade	154 (0.9%)	156 (0.9%)	−0.1%	34 (0.2%)	37 (0.3%)	−0.4%
High school diploma	6641 (36.9%)	6436 (35.7%)	2.4%	3801 (26.6%)	3871 (27.1%)	−1.1%
Less than a bachelor's degree	8663 (48.1%)	8882 (49.3%)	−2.4%	7442 (52.0%)	7420 (51.9%)	0.3%
Bachelor's degree plus	1361 (7.6%)	1386 (7.7%)	−0.5%	2133 (14.9%)	2135 (14.9%)	−0.0%
Unknown	1196 (6.6%)	1155 (6.4%)	0.9%	895 (6.3%)	842 (5.9%)	1.6%
**Clinical characteristics**						
**CCI, mean (SD)**	2.40 (2.1)	2.27 (2.1)	6.4%	1.23 (1.7)	1.19 (1.7)	2.2%
CCI comorbidities, *n* (%)						
AIDS/HIV	60 (0.3%)	68 (0.4%)	−0.7%	42 (0.3%)	48 (0.3%)	−0.7%
Any malignancy (excl. malignant neoplasm of skin)	1691 (9.4%)	1618 (9.0%)	1.4%	1249 (8.7%)	1223 (8.5%)	0.6%
Cerebrovascular disease	3535 (19.6%)	3287 (18.2%)	3.5%	1397 (9.8%)	1216 (8.5%)	4.4%
Chronic pulmonary disease^c^	6136 (34.1%)	5929 (32.9%)	2.4%	4004 (28.0%)	4199 (29.4%)	−3.0%
Congestive heart failure	3912 (21.7%)	3444 (19.1%)	6.4%	1072 (7.5%)	883 (6.2%)	5.2%
Dementia	691 (3.8%)	554 (3.1%)	4.2%	228 (1.6%)	169 (1.2%)	3.5%
Diabetes with chronic complication^d^	9045 (50.2%)	9089 (50.5%)	−0.5%	0 (0.0%)	0 (0.0%)	0
Diabetes without chronic complication^d^	7705 (42.8%)	7851 (43.6%)	−1.6%	7 (<0.1%)	2 (<0.1%)	2.0%
Hemiplegia or paraplegia	483 (2.7%)	413 (2.3%)	2.5%	232 (1.6%)	178 (1.2%)	3.2%
Metastatic solid tumor	276 (1.5%)	275 (1.5%)	0.0%	251 (1.8%)	222 (1.6%)	1.6%
Mild liver disease	2582 (14.3%)	2497 (13.9%)	1.4%	1791 (12.5%)	1983 (13.9%)	−4.0%
Moderate or severe liver disease	296 (1.6%)	250 (1.4%)	2.1%	121 (0.8%)	109 (0.8%)	0.9%
Myocardial infarction	1882 (10.4%)	1689 (9.4%)	3.6%	542 (3.8%)	467 (3.3%)	2.8%
Peptic ulcer disease	1006 (5.6%)	805 (4.5%)	5.1%	944 (6.6%)	771 (5.4%)	5.1%
Peripheral vascular disease	4325 (24.0%)	4058 (22.5%)	3.5%	1511 (10.6%)	1301 (9.1%)	4.9%
Renal disease	5161 (28.6%)	4790 (26.6%)	4.6%	1107 (7.7%)	903 (6.3%)	5.6%
Rheumatic disease	1186 (6.6%)	1126 (6.3%)	1.4%	1114 (7.8%)	1071 (7.5%)	1.1%
**Diabetes characteristics, *n* (%)** ^e^						
Diabetes type						
Type I diabetes	223 (1.2%)	188 (1.0%)	1.8%			
Type II diabetes	11,856 (65.8%)	12,043 (66.8%)	−2.2%			
Unknown diabetes type	4665 (25.9%)	4709 (26.1%)	−0.6%			
Overall insulin users	7598 (42.2%)	7652 (42.5%)	−0.6%	–	–	
Diabetic complications						
Angina/heart failure	1328 (7.4%)	1283 (7.1%)	1.0%	–	–	
Diabetic foot	2083 (11.6%)	2055 (11.4%)	0.5%	–	–	
Hypertension	15,348 (85.2%)	15,684 (87.1%)	−5.4%	–	–	
Nephropathy	2924 (16.2%)	2889 (16.0%)	0.5%	–	–	
Neuropathy	6120 (34.0%)	6041 (33.5%)	0.9%	–	–	
Obesity	4733 (26.3%)	4627 (25.7%)	1.3%	–	–	
Retinopathy	3679 (20.4%)	3716 (20.6%)	−0.5%	–	–	
Stroke	155 (0.9%)	156 (0.9%)	−0.1%			

OTHER VARIABLES						
Comorbidities more commonly seen in patients with gastroparesis, *n* (%)	DG cohort	Matched controls	*p*	IG cohort	Matched controls	*p*
Anemia	6813 (37.8%)	5387 (29.9%)	<0.0001*	3407 (23.8%)	2092 (14.6%)	<0.0001*
Anxiety disorder	4249 (23.6%)	2675 (14.8%)	<0.0001*	4076 (28.5%)	2090 (14.6%)	<0.0001*
Asthma	2991 (16.6%)	2669 (14.8%)	<0.0001*	2227 (15.6%)	2147 (15.0%)	0.1153
Cardiac arrhythmias	4158 (23.1%)	3505 (19.5%)	<0.0001*	2364 (16.5%)	1515 (10.6%)	<0.0001*
Chronic pain syndrome	1376 (7.6%)	738 (4.1%)	<0.0001*	862 (6.0%)	273 (1.9%)	<0.0001*
Chronic pancreatitis	825 (4.6%)	317 (1.8%)	<0.0001*	558 (3.9%)	125 (0.9%)	<0.0001*
Depression	5396 (30.0%)	3779 (21.0%)	<0.0001*	4040 (28.2%)	2313 (16.2%)	<0.0001*
Epilepsy	573 (3.2%)	394 (2.2%)	<0.0001*	420 (2.9%)	228 (1.6%)	<0.0001*
ESRD	958 (5.3%)	610 (3.4%)	<0.0001*	95 (0.7%)	56 (0.4%)	0.0015*
Fibromyalgia	2032 (11.3%)	1305 (7.2%)	<0.0001*	1984 (13.9%)	947 (6.6%)	<0.0001*
Functional dyspepsia	2532 (14.1%)	280 (1.6%)	<0.0001*	3533 (24.7%)	257 (1.8%)	<0.0001*
Hypothyroidism	4869 (27.0%)	4247 (23.6%)	<0.0001*	2943 (20.6%)	2177 (15.2%)	<0.0001*
Irritable bowel syndrome	1088 (6.0%)	342 (1.9%)	<0.0001*	1674 (11.7%)	315 (2.2%)	<0.0001*
Migraine	1044 (5.8%)	490 (2.7%)	<0.0001*	1562 (10.9%)	578 (4.0%)	<0.0001*
Obstructive sleep apnea	3051 (16.9%)	2412 (13.4%)	<0.0001*	1171 (8.2%)	680 (4.8%)	<0.0001*
Paralytic ileus	437 (2.4%)	136 (0.8%)	<0.0001*	383 (2.7%)	80 (0.6%)	<0.0001*
Systemic lupus erythematosus	384 (2.1%)	292 (1.6%)	0.0003*	534 (3.7%)	344 (2.4%)	<0.0001*
Venous thromboembolism	475 (2.6%)	388 (2.2%)	0.0027*	272 (1.9%)	151 (1.1%)	<0.0001*
**All‐cause HRU, *n* (%)**						
Patients with inpatient admission(s)	8312 (46.1%)	6307 (35.0%)	<0.0001*	4461 (31.2%)	2738 (19.1%)	<0.0001*
Patients with ER visit(s)	11,002 (61.1%)	7971 (44.2%)	<0.0001*	7102 (49.6%)	4204 (29.4%)	<0.0001*
Patients with outpatient visit(s)	14,908 (82.8%)	13,030 (72.3%)	<0.0001*	12,372 (86.5%)	9244 (64.6%)	<0.0001*
**All‐cause health‐care cost per year (2019 USD)**						
Total cost, mean (SD)	$52,570 ($105,062)	$35,919 ($83,559)	<0.0001*	$33,704 ($80,782)	$16,242 ($38,357)	<0.0001*

Notes

Comparisons between matched DG and control patients and between matched IG and control patients were conducted using Wilcoxon signed‐rank test for continuous variables and McNemar's test for categorical variables.

Abbreviations: AIDS, acquired immunodeficiency syndrome; CCI, Charlson Comorbidity Index; DG, diabetic gastroparesis; ER, emergency room; ESRD, end‐stage renal disease; HIV, human immunodeficiency virus; HRU, health‐care resource use; IG, idiopathic gastroparesis; SD, standard deviation; USD, US dollars.

^a^ Standardized mean differences (in absolute value) below 10% indicate that propensity matching achieved its purpose of balancing the baseline covariates between the matched cohorts. Variables that were exactly matched are denoted "─".

^b^ Cases with ≥3 years of continuous enrollment after index date were matched exactly to controls with ≥3 years of continuous enrollment after the index date. Cases with ≥2 years of continuous enrollment after index date were matched exactly to controls with ≥2 years of continuous enrollment after the index date, which were allowed to also have ≥3 years of continuous enrollment after the index date.

^c^ Under the CCI, chronic pulmonary disease includes a broad list of diseases such as asthma, chronic obstructive pulmonary disease, chronic pulmonary heart diseases, emphysema, bronchiectasis, bronchitis, and other conditions identified via diagnosis codes.

^d^ A minority of patients had diabetes diagnosis codes before the index date but not in the baseline period. The baseline table captured diabetes diagnosis codes during the baseline period.

^e^ Only applicable to DG cohort. A minority of patients had diagnosis codes for diabetes before the index date but not during the baseline period. The baseline table captured diagnosis codes for diabetes during the baseline period (the 1 year prior to the index date). Patients with unknown diabetes type had diagnosis codes for both Type I diabetes and Type 2 diabetes during the baseline period.

* *p* < 0.05.

All‐cause HRU outcomes included inpatient admissions and total inpatient days, and ER, outpatient, and other health‐care use (e.g., durable medical equipment, transportation services, and other professional services such as diagnostic testing, laboratory, or radiology). All‐cause total cost outcomes included all‐cause costs (i.e., estimated claims paid) for pharmacy and medical services (inpatient, ER, outpatient, and other visits). Health‐care costs were adjusted to 2019 US dollars (USD) using the medical care component of the Consumer Price Index.^33^

### 2.4 Statistical analysis

Baseline characteristics including demographics as of the index date and comorbidities, HRU, and costs in the year prior to the index date were described using means, standard deviations (SD), frequencies, and percentages. For patient characteristics in the propensity score models, standardized mean differences between patients with DG/IG and their respective matched controls were used to evaluate the matching performance (typically, absolute value of standardized differences within 10% is perceived as balanced).^34^ For all other characteristics, comparisons were conducted using Wilcoxon signed‐rank test for continuous variables and McNemar's test for categorical variables.

This study compared the all‐cause HRU and associated costs among patients with a gastroparesis diagnosis to that of the matched controls from the general population, and the difference in the burden was assumed attributable to gastroparesis. All‐cause HRU (proportions of patients with admissions/visits and means of admissions/visits) and all‐cause health‐care costs (means and SDs) were described among the matched pairs during the 1st, 2nd, and 3rd year post‐index date. HRU comparisons between the DG/IG cohorts and their respective matched controls were conducted using Wilcoxon signed‐rank test for mean admissions/visits and McNemar's test for proportions of patients with admissions/visits, while cost comparisons were conducted using Wilcoxon signed‐rank test.

Over the up to 3‐year follow‐up period, HRU incidence rate ratios (IRR) were estimated to compare the incidence rates of admission/visit occurrence using generalized estimating equations (GEE) models with binomial distribution and repeated HRU measurements (one HRU measurement for each year with complete follow‐up post‐index). GEE models with Tweedie distribution were used to estimate cost differences over the follow‐up period. In both cases, the GEE methodology accounted for the repeated measures within subjects over the 3‐year follow‐up and for the matched design.

### 2.5 Sensitivity analyses

In the sensitivity analyses, the regression models for HRU and costs were further adjusted for comorbidities more commonly seen in patients with gastroparesis^32^ (unmatched variables), total all‐cause baseline costs (unmatched variables), and index year (matched variable, but adjusted in the model because the standardized difference was >|0.10| post‐matching). Because this approach further removes the pre‐index differences between cohorts that may reflect the burden of gastroparesis pre‐diagnosis (e.g., anemia, functional dyspepsia, or anxiety disorder^32^), the estimates are expected to be conservatively low.

## 3 RESULTS

### 3.1 Patients with DG versus matched diabetic controls

After meeting all eligibility criteria, 18,015 patients with DG were matched to diabetic controls (i.e., without gastroparesis and with ≥1 year of follow‐up post‐index) and included in the study (Figure 1A).

#### 3.1.1 Baseline characteristics

Among patients with DG, the mean age was 62 years, 66% were female, and 55% were white (Table 1). On average, patients with DG had a CCI of 2.4, and the top three most common CCI comorbidities beyond diabetes were chronic pulmonary disease (34%), renal disease (29%), and peripheral vascular disease (24%). Other prevalent comorbidities in patients with DG (i.e., ≥25% prevalence) included anemia (38%), depression (30%), and hypothyroidism (27%).

All matching variables were well balanced between the patients with DG and their matched controls (standardized differences <10% for all, with the exception of index year in 2016–2018, which had a standardized difference of |0.171| and was further adjusted in the sensitivity analysis).

Comorbidities that may reflect the burden of gastroparesis before the formal diagnosis (unmatched) were significantly more frequent among the DG cohort at baseline compared with the matched controls **(**all *p* < 0.05**)**. The largest differences in proportions between the DG cohort and the matched controls were observed for functional dyspepsia (14% vs. 2%) and depression (30% vs. 21%).

All‐cause HRU and costs at baseline were significantly higher for the DG cohort compared to matched controls (all *p* < 0.001) (Table 1). Total all‐cause health‐care costs in the year before the index date were $52,570 (SD: $105,062) for the DG cohort and $35,919 (SD: $83,559; *p* < 0.001) for the matched controls.

#### 3.1.2 Outcomes

**FIGURE 2:**
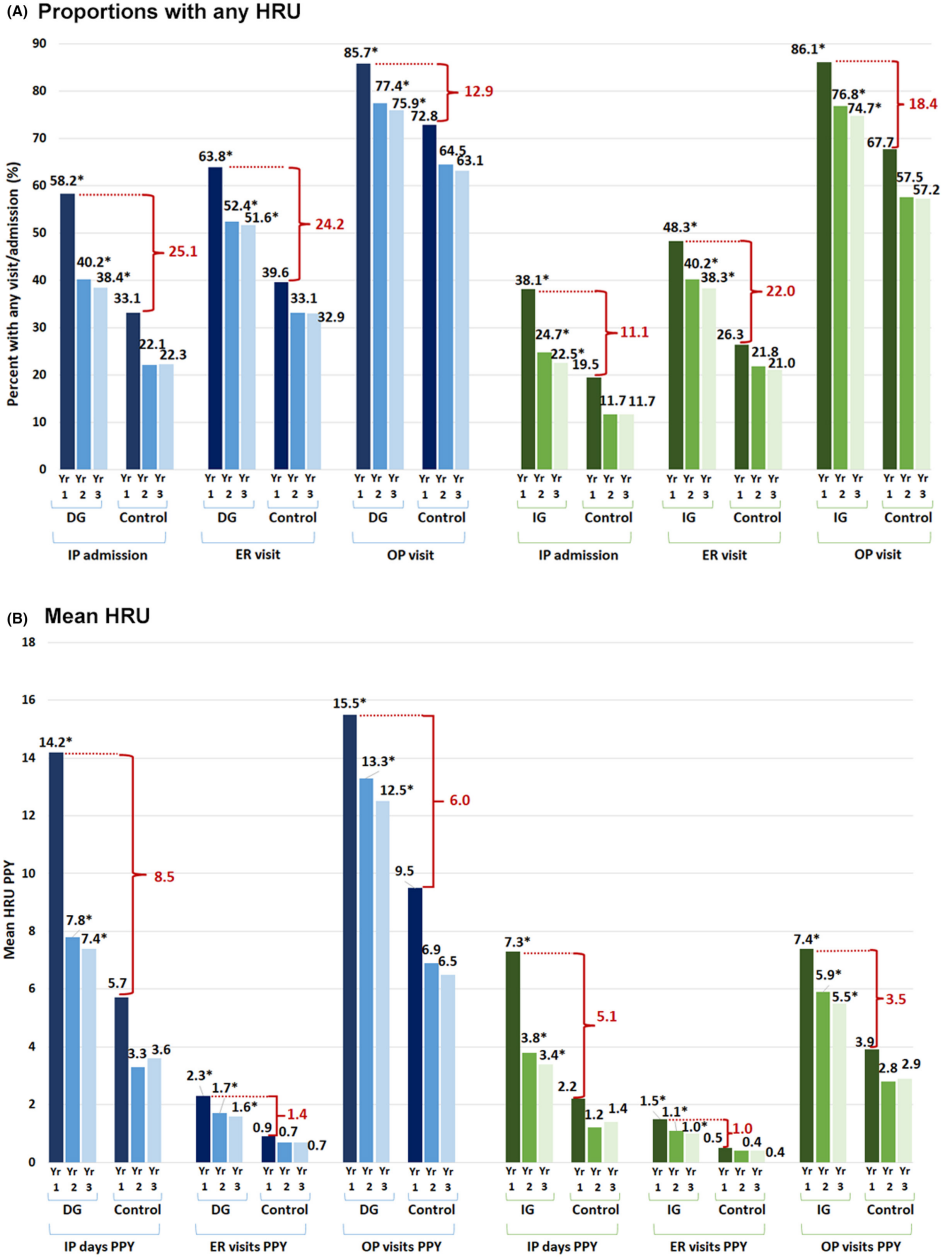
Proportions with any HRU (A) and mean HRU (B) in the First 3 Years Post‐Gastroparesis Diagnosis for the DG and IG Cohorts versus Matched Gastroparesis‐free Controls. Abbreviations: DG, diabetic gastroparesis; ER, emergency room; GP, gastroparesis; HRU, health‐care resource use; IG, idiopathic gastroparesis; IP, inpatient; OP, outpatient; Yr, year. *p < 0.001 for comparisons to matched controls in the same year post‐GP diagnosis for the same HRU measure

All DG matched pairs (18,015) were included in the Year 1 analysis. For the Year 2 and Year 3 analyses, 11,870 and 7838 DG‐matched pairs, respectively, had applicable follow‐up time to be included. Compared to the matched controls, the DG cohort had significantly higher proportions experiencing any HRU (Figure 2A), and significantly higher mean HRU (Figure 2B), in each year of the 3‐year follow‐up period. The DG cohort incurred the highest mean HRU in the 1st year after the initial diagnosis of gastroparesis (mean annual HRU vs. matched controls in Year 1: 14.2 vs. 5.7 inpatient days, 2.3 vs. 0.9 ER visits, and 15.5 vs. 9.5 outpatient visits; all *p* < 0.001). Over the 3‐year follow‐up period, patients with DG had significantly higher rates of inpatient admissions (IRR: 1.89), ER visits (2.33), outpatient visits (1.73), and other admissions/visits (1.46), and significantly more total inpatient days (2.21), than their matched controls (all *p* < 0.001; Table 2).

**TABLE 2:** Comparison of HRU and costs for the DG and IG cohorts and matched controls during the 3‐year follow‐up period

	DG cohort versus matched controls		IG cohort versus matched controls	
	During the 3‐Year Follow‐up Period^a^, ^b^			
All‐cause HRU	Incidence rate ratio	*p*	Incidence rate ratio	*p*
Number of visits or admissions per year				
IP admissions	1.89	<0.001*	2.47	<0.001*
IP days	2.21	<0.001*	2.89	<0.001*
ER visits	2.33	<0.001*	2.83	<0.001*
OP visits	1.73	<0.001*	1.94	<0.001*
Other admissions/visits	1.46	<0.001*	1.79	<0.001*

All‐cause health‐care costs per year (2019 USD)	Mean annual cost difference	*p*	Mean annual cost difference	*p*
Medical costs	$22,907	<0.001*	$13,759	<0.001*
IP admissions	$9283	<0.001*	$5884	<0.001*
ER visits	$2300	<0.001*	$1806	<0.001*
OP visits	$7700	<0.001*	$3272	<0.001*
Other admissions/visits	$3588	<0.001*	$2806	<0.001*
Pharmacy costs	$1552	<0.001*	$1716	<0.001*
Total costs	$24,465	<0.001*	$15,479	<0.001*

Abbreviations: DG, diabetic gastroparesis; ER, emergency room; HRU, health‐care resource use; IG, idiopathic gastroparesis; IP, inpatient; OP, outpatient; SD, standard deviation; USD, United States dollars.

*p < 0.001.

^a^ Results for the 3‐year follow‐up period were estimated using generalized estimating equations accounting for the repeated measures within subjects over 3 years and the matched design.

^b^ Patients with complete follow‐up for the 1st, 2nd, and 3rd years after the index date contributed to the analysis of the 3‐year follow‐up period. For DG, 18,015 patients and matched controls contributed to the analysis for Year 1, 11,870 contributed to both Year 1 and Year 2, and 7838 contributed to all 3 years. For IG, 14,305 patients and matched controls contributed to the analysis for Year 1, 9857 contributed to both Year 1 and Year 2, and 6837 contributed to all 3 years.

**FIGURE 3:**
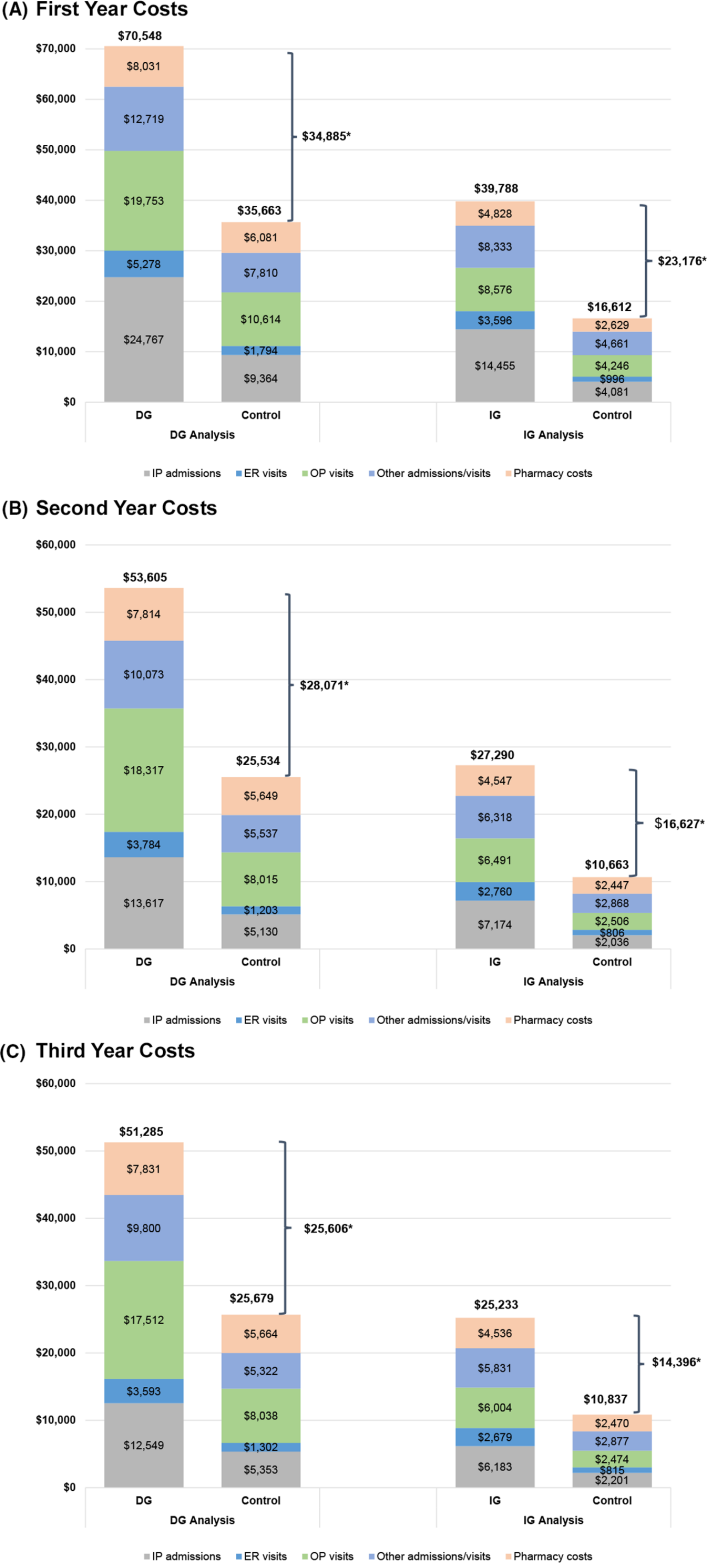
Mean Health‐care Costs of the DG and IG Cohorts in the (A) 1st, (B) 2nd, and (C) 3rd years Post‐Gastroparesis Diagnosis (2019 USD), versus Matched Gastroparesis‐free Controls. Abbreviations: DG, diabetic gastroparesis; ER, emergency room; IG, idiopathic gastroparesis; IP, inpatient; OP, outpatient; USD, US dollars. *p < 0.001 for comparisons to matched controls in the same year post‐gastroparesis diagnosis for the cost measure

Health‐care costs measured during each of the 3 years of follow‐up were all significantly higher among the gastroparesis cohorts compared with their respective matched controls (Figure 3A–C). For the DG cohort, total costs were $70,548 in the 1st year, $53,605 in the 2nd year, and $51,285 in the 3rd year; for the matched controls, total costs were $35,663, $25,534, and $25,679, respectively. In addition, patients with DG had significantly higher total costs per year than matched controls (cost difference: $24,465), largely due to differences in medical costs ($22,907) driven by inpatient costs ($9283) and outpatient costs ($7700; all *p* < 0.001) (Table 2).

### 3.2 Patients with IG versus matched non‐diabetic controls

After meeting all eligibility criteria, 14,305 IG‐matched pairs were included in the study (Figure 1B). By design, all of these patients had at least 1 year of follow‐up post‐index.

#### 3.2.1 Baseline characteristics

Among patients with IG, the mean age was 54 years, mean CCI was 1.2, 76% were female, and 65% were white (Table 1). The top three most common CCI comorbidities were chronic pulmonary disease (28%), mild liver disease (13%), and peripheral vascular disease (11%). Other prevalent comorbidities (i.e., ≥25% prevalence) included anxiety disorder (29%), depression (28%), and functional dyspepsia (25%).

All characteristics matched between the patients with IG and controls were well balanced (absolute value of standardized differences <10% as balanced with the exception of index year in 2016–2018, which had a standardized difference of |0.101| and was further adjusted in the sensitivity analysis).

Most comorbidities that may reflect the burden of gastroparesis before the formal diagnosis (i.e., comorbidities more often seen in patients with gastroparesis,^32^ as listed in Table 1) were significantly more frequent among the IG cohort at baseline compared with the matched controls **(** *p* < 0.05**)**. The three largest differences in proportions between the IG cohort and matched controls were functional dyspepsia (25% vs. 2%), anxiety disorder (29% vs. 15%), and depression (28% vs. 16%). All‐cause HRU and costs at baseline were significantly higher for the IG cohort than their respective matched controls (all *p* < 0.001) (Table 1). Total all‐cause health‐care costs in the year before the index date were $33,704 (SD: $80,782) for the IG cohort and $16,242 (SD: $38,357; *p* < 0.001) for the matched controls.

#### 3.2.2 Outcomes

All IG‐matched pairs (14,305) were included in the Year 1 analysis. For the Year 2 and Year 3 analyses, 9857 and 6837 IG‐matched pairs, respectively, had applicable follow‐up time to be included. The IG cohort had significantly higher proportions experiencing any HRU (Figure 2A) versus controls, and significantly higher mean HRU (Figure 2B), in each year of the 3‐year follow‐up period. The IG cohort incurred the highest mean HRU in the 1st year after gastroparesis diagnosis (mean annual HRU vs. matched controls in Year 1: 7.3 vs. 2.2 inpatient days, 1.5 vs. 0.5 ER visits, and 7.4 vs. 3.9 outpatient visits; all *p* < 0.001). Over the 3‐year follow‐up period, patients with IG had significantly higher annual rates of inpatient admissions (IRR: 2.47), ER visits (2.83), outpatient visits (1.94), and other admissions/visits (1.79), and significantly more total inpatient days (2.89), than matched controls (all *p* < 0.001; Table 2).

Health‐care costs measured during each of the 3 years of follow‐up were all significantly higher among the IG cohort compared with matched controls (Figure 3A–C). For the IG cohort, mean total costs were $39,788 in the 1st year, $27,290 in the 2nd year, and $25,233 in the 3rd year; the controls’ health‐care costs were $16,612, $10,663, and $10,837, respectively. Patients with IG had significantly higher total costs per year (cost differences: $15,479) versus controls, largely due to differences in medical costs ($13,759; both *p* < 0.001). Among medical costs, the largest cost differences were observed for inpatient costs (cost differences: $5884), followed by outpatient costs ($3272) (both *p* < 0.001; Table 2).

### 3.3 Sensitivity analysis

The findings from the sensitivity analysis were consistent with those of the main analyses (Table S1). After statistical adjustment for several characteristics that were unbalanced at baseline, patients with DG or IG continued to have significantly higher HRU and costs compared with matched controls. Specifically, for the DG cohort, the IRRs were 1.78 for inpatient admissions, 1.96 for ER visits, and 1.55 for outpatient visits, with a total cost difference of $19,833 (all *p* < 0.001). For the IG cohort, the IRRs were 1.73 for inpatient admissions, 1.81 for ER visits, and 1.42 for outpatient visits, with a total cost difference of $8482 (all *p* < 0.001).

## 4 DISCUSSION

Gastroparesis may be associated with debilitating symptoms, but few real‐world studies have quantified the associated economic burden using population‐level data. To our knowledge, this large real‐world study is the first assessment of the longitudinal HRU and related costs across multiple care settings following the initial gastroparesis diagnosis. The results in each of the 3 years after the initial gastroparesis diagnosis indicated that patients with DG or IG incurred significantly more annual all‐cause HRU and higher health‐care costs than their matched controls, with the largest economic burden observed in the 1^st^ year. For both the DG and IG cohorts, this finding was consistent across all service settings (inpatient, ER, outpatient, and other admissions/visits) and cost measures (total costs and the component medical and pharmacy costs).

Prior analyses of the economic burden of gastroparesis have generally focused on limited health‐care settings, primarily inpatient, or used data other than claims.^16^, ^17^, ^26^ Lacy et al.^17^ used a survey to determine the hospitalization rate due to gastroparesis among patients with gastroparesis symptoms (50% in the year prior to the survey). Wadhwa et al.^16^ used the National Inpatient Sample database to determine the mean US hospital charge among patients discharged with a principal diagnosis of gastroparesis ($34,585 per patient [2013 USD]). Hirsch et al.^26^ used the US Nationwide Emergency Department Sample database to estimate the mean charges per gastroparesis‐related ER visit $4352 and inpatient admission $32,563 (2013 USD). The HRU and cost estimates from the present economic burden study are not directly comparable to these studies due to the differences in patient selection, burden estimation method (e.g., cost difference between gastroparesis patients and matched controls in all‐cause services vs. cost only for gastroparesis‐specific services), and length of follow‐up (longitudinal follow‐up for up to 3 years after the gastroparesis diagnosis vs. one or multi‐year snapshots among patients with a prior diagnosis of gastroparesis). Due to the underdiagnosis of gastroparesis and undercapturing of gastroparesis‐related encounters in the claims database,^32^ the complete burden of gastroparesis tends to be underestimated. To mitigate such underestimation, this study compared all‐cause HRU and associated costs among patients with a gastroparesis diagnosis versus that of the matched controls from the general population, and the difference between the matched cohorts was burden attributable to gastroparesis. The present results, along with those of prior analyses, collectively quantify the substantial economic burden of gastroparesis.

In the present study, the highest HRU and costs for the DG and IG cohorts were observed in the 1st year after gastroparesis diagnosis, largely attributable to higher inpatient costs. There are several potential explanations. This finding may be related to the timing of the initial diagnosis if it occurred during a health crisis or flare‐up of gastroparesis symptoms, leading to more costly HRU (i.e., ER visits, inpatient admissions, and diagnostic testing) and accordingly higher spending. At the initial gastroparesis diagnosis, a number of diagnostic tests (e.g., esophagogastroduodenoscopy and gastric emptying test) are typically performed, which may not be repeated in the following years. While the HRU and costs for controls were also highest in Year 1, so was the difference in the economic burden between the controls and the DG/IG cohorts. This supports the perception that higher Year 1 costs and HRU for the DG and IG cohorts were likely due to a flare‐up in gastroparesis symptoms or diagnostic testing. In addition, the 1^st^ year following diagnosis is when treating physicians are trying to find the appropriate disease management for a patient, which may lead to more frequent follow‐up visits during that process. Another possibility is that the patients with severe symptoms or the very ill—who would be likely to have the costliest HRU—were lost to follow‐up in Years 2 and 3 of the analysis. However, although the HRU and costs were lower in Years 2 and 3 for the DG and IG cohorts, the overall burden of gastroparesis remained twice as high in those years compared to matched controls. This consistently high economic burden of gastroparesis after initial diagnosis and treatment could be related to the lack of effective treatment options that safely manage its chronic symptoms.^14^, ^15^

The suboptimal management options for gastroparesis symptoms may result in short treatment durations and frequent therapy switching. A recent study found that patients newly diagnosed with gastroparesis often remain untreated for extended periods after an initial attempt with conventional therapies, including metoclopramide and other treatments used in real‐world practice.^35^ In general, treatment duration is brief during the 1st year following gastroparesis diagnosis and 39%–57% of patients switched therapies in the first few months,^35^ indicating poor symptom control or tolerability issues. Indeed, 60% of patients with gastroparesis in a 2017 survey expressed dissatisfaction with their therapy.^25^ The burden of persistent gastroparesis symptoms^16^, ^36^, ^37^, ^38^ and its negative impact on HRQoL and work productivity,^17^ as described in prior studies, also indicate the suboptimal performance of current treatments. Multiple studies have reported clinically assessed prevalence of persistent gastroparesis symptoms, including nausea and vomiting (up to 58% of patients),^36^ abdominal pain (up to 51%),^16^, ^36^, ^38^ and diarrhea and constipation (around 17%).^37^ Other studies of self‐reported outcomes have confirmed the high symptom burden of gastroparesis, with 85–95% of patients reporting nausea and 25–45% reporting abdominal pain,^39^, ^40^, ^41^, ^42^ and high rates of total parenteral nutrition (~20% in the prior year) or a feeding tube (19%),^17^ and reduction in daily activities, school/work schedules, and annual income.^17^ Patients with gastroparesis also experience frequent 30‐ and 90‐day inpatient readmissions, estimated at 26.8–35% and 45.6%, respectively.^43^, ^44^ The indirect costs of gastroparesis, and the subsequent loss of income or opportunity, further contribute to the overall disease burden to patients and society. Furthermore, a rising incidence of gastroparesis diagnoses (both IG and DG), and hospitalizations and ER visits related to gastroparesis, has been reported over different time periods from 1997 to 2013.^16^, ^26^ This rise in incidence raises concern for escalating costs to the health‐care system related to gastroparesis.

This study has several strengths in design and data. First, the use of a large US claims database permitted capturing a much larger sample size than previous studies. This also allowed the identification of suitable controls without gastroparesis from the general population to assess the disease‐attributable burden, and among subpopulations (e.g., IG and DG). The use of matched controls mitigated potential confounding, as the matched baseline characteristics were comparable and well balanced between cases and controls. In addition, to mitigate the challenge that gastroparesis‐related claims may not be fully reflected in the claims database, a method for assessing the total disease burden was employed, by quantifying the differences in all‐cause HRU and costs between matched cases and controls. Second, the present methodology, using a robust design of propensity score matching, permitted adjustments to demographic and clinical characteristics that differed between the gastroparesis case cohorts and their matched controls. Potential confounding was mitigated by matching in the main analyses, while the sensitivity analyses controlled for additional baseline characteristics and provided a conservative estimate to reflect the lower bound of gastroparesis burden. The controls that were matchable with the cases were at higher risk than the average person within the general population; thus, the disease burden of gastroparesis quantified in this study is a conservative estimate. Finally, this study quantified the etiology‐specific economic burden associated not only with DG but also with IG, a subgroup of gastroparesis with scant real‐world evidence reported in the literature. Prior studies have focused more on the overall patient population with gastroparesis or patients with DG.

This study is also subject to a few limitations. First, as with any retrospective claims database, occasional miscoding may occur in the claims data. Furthermore, as patients may change insurance plans over years, it could be possible that some patients were previously diagnosed with gastroparesis but not reflected in this claims database. Second, gastroparesis is often underdiagnosed (e.g., with delay) or misdiagnosed (e.g., as functional dyspepsia^23^) in real‐world practice. This may result in the underestimation of the burden of gastroparesis in this study and also may explain the high prevalence of baseline comorbidities related to gastroparesis among newly diagnosed patients. Thus, a portion of the burden of gastroparesis (i.e., diagnostic tests and procedures) may occur prior to the initial diagnosis, which was managed via analytical methods by assessing costs and HRU during the 1 year prior to the index date (baseline period). The sensitivity analyses adjusting for additional baseline comorbidities and health‐care costs provided an estimate which is likely lower than the true burden of gastroparesis. Third, these results primarily apply to patients covered by commercial health insurance and may not represent patients with other types of insurance or the uninsured. Fourth, the parameters used when defining the IG cohort, which is the residual category after the assignment of other etiologies, may not be exhaustive. Fifth, there are several limitations related to the identification of patients with diabetes using the Optum claims data. Type I and type II diabetes were identified in the database using diagnosis codes, which could be subject to potential misclassification and coding errors. We classified approximately 25.9% of DG patients with unknown diabetes type because they had codes for both type I and type II diabetes during the baseline period. In addition, insulin use was calculated based only on the 1‐year baseline period and among the patients with diabetes who met the sample selection criteria, which may undercapture patients’ insulin use history. The relative risks of the occurrence of diabetic complications by type I or type II diabetes were not assessed in this economic burden study. Lastly, the results may include bias due to unobserved factors that could not be adjusted for as covariates in the matched design.

In conclusion, this retrospective US claims study demonstrates the substantial economic burden associated with DG and IG over each of the first 3 years after the initial diagnosis of gastroparesis. This increased economic burden is reflected by patients’ greater comorbidity burden and higher all‐cause HRU and health‐care costs compared with matched controls. The matched design permitted the ability to determine the difference in HRU and costs attributable to gastroparesis. The difference in the economic burden experienced by patients with DG or IG versus their matched controls was the highest in the 1st year after gastroparesis diagnosis and remained significantly higher than that of controls over all 3 years of follow‐up. These findings emphasize the need for gastroparesis therapies suitable for chronic symptom and disease management, which may reduce the burden of illness and improve the HRQoL of patients with gastroparesis.

## CONFLICT OF INTEREST

YJC and SYH are employees of Takeda Pharmaceuticals and own stock and/or options. WT, RA, and EW are employed by Analysis Group, Inc. which received funding from Takeda to conduct this study; RI was employed by Analysis Group, Inc. during the study's conduct. HPP is a consultant for Takeda.

## AUTHOR CONTRIBUTIONS

All authors designed the research study, performed the research, and contributed to drafting and editing the paper. WT, RA, RI, and EW analyzed the data.

## Supporting information

Supplementary Material
