# Supplementary material for: Health‐care resource use and costs associated with diabetic and idiopathic gastroparesis: A claims analysis of the first 3 years following the diagnosis of gastroparesis
Source: Neurogastroenterol Motil. 2022 Mar 30;34(9):e14366. doi: 10.1111/nmo.14366 (PMC9539633; doi:10.1111/nmo.14366)
Supplement: Supplementary file 1 — Supplementary Material [file NMO-34-e14366-s001.docx]

**SUPPLEMENTAL MATERIALS**

**Supplemental Table 1. Sensitivity Analysis**


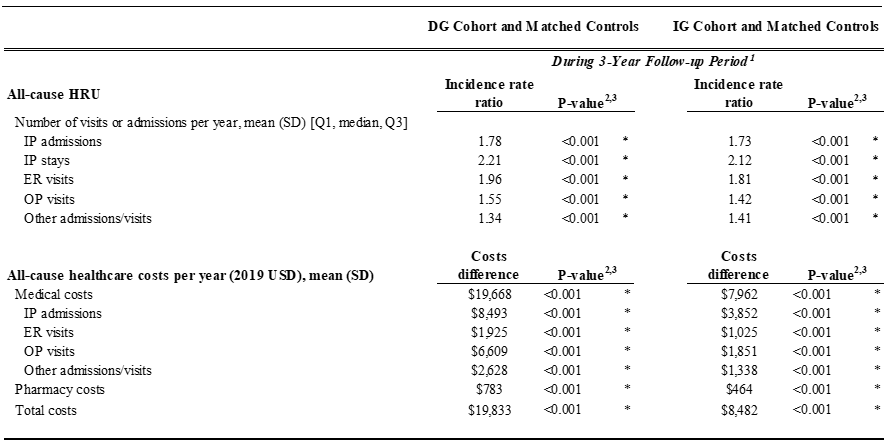


**Abbreviations:** CI = confidence interval, DG = diabetic gastroparesis, ER = emergency room, GEE = generalized estimating equation, HRU = healthcare resource use, IG = idiopathic gastroparesis, IP = inpatient, OP = outpatient, Q = quarter, SD = standard deviation, USD = United States dollars

**Notes:**

^1^ In the sensitivity analysis, incidence rate ratios, 95% CIs, and p-values for mean HRU were estimated using multivariable GEE models with negative binomial distribution, and cost differences and p-values for proportions were estimated using GEE models with Tweedie distribution. The sensitivity analyses controlled for all comorbidities more commonly seen in patients with gastroparesis (full list can be found in **Table 1**; defined based on literature), the index year, and total costs in the baseline period.

^2^ Results for the 3-year follow-up period were estimated using GEEs accounting for the repeated measures within subjects over 3 years and the matched design.

^3^ Patients with complete follow-up for the first, second, and third years after the index date contributed to the analysis of the 3-year follow-up period. For DG, 18,015 patients and matched controls contributed to the analysis for Year 1; 11,870 contributed to both Year 1 and Year 2; and 7,838 contributed to all three years. For IG, 14,305 patients and matched controls contributed to the analysis for Year 1; 9,857 contributed to both Year 1 and Year 2; and 6,837 contributed to all three years.
